# Reciprocal interaction between mitochondrial fission and mitophagy in postoperative delayed neurocognitive recovery in aged rats

**DOI:** 10.1111/cns.14261

**Published:** 2023-05-19

**Authors:** Yitong Li, Yue Li, Lei Chen, Yi Li, Kaixi Liu, Jingshu Hong, Qian Wang, Ning Kang, Yanan Song, Xinning Mi, Yi Yuan, Dengyang Han, Taotao Liu, Ning Yang, Xiangyang Guo, Zhengqian Li

**Affiliations:** ^1^ Department of Anesthesiology Peking University Third Hospital Beijing China; ^2^ Department of Anesthesiology Beijing Jishuitan Hospital Beijing China

**Keywords:** aged rats, delayed neurocognitive recovery, mitochondrial dysfunction, mitochondrial fission, mitophagy

## Abstract

**Introduction:**

Emerging evidence suggests that mitochondrial dysfunction plays a crucial role in the pathogenesis of postoperative delayed neurocognitive recovery (dNCR). Mitochondria exist in a dynamic equilibrium that involves fission and fusion to regulate morphology and maintains normal cell function via the removal of damaged mitochondria through mitophagy. Nonetheless, the relationship between mitochondrial morphology and mitophagy, and how they influence mitochondrial function in the development of postoperative dNCR, remains poorly understood. Here, we observed morphological alterations of mitochondria and mitophagy activity in hippocampal neurons and assessed the involvement of their interaction in dNCR following general anesthesia and surgical stress in aged rats.

**Methods:**

Firstly, we evaluated the spatial learning and memory ability of the aged rats after anesthesia/surgery. Hippocampal mitochondrial function and mitochondrial morphology were detected. Afterwards, mitochondrial fission was inhibited by Mdivi‐1 and siDrp1 in vivo and in vitro separately. We then detected mitophagy and mitochondrial function. Finally, we used rapamycin to activate mitophagy and observed mitochondrial morphology and mitochondrial function.

**Results:**

Surgery impaired hippocampal‐dependent spatial learning and memory ability and caused mitochondrial dysfunction. It also increased mitochondrial fission and inhibited mitophagy in hippocampal neurons. Mdivi‐1 improved mitophagy and learning and memory ability of aged rats by inhibiting mitochondrial fission. Knocking down Drp1 by siDrp1 also improved mitophagy and mitochondrial function. Meanwhile, rapamycin inhibited excessive mitochondrial fission and improved mitochondrial function.

**Conclusion:**

Surgery simultaneously increases mitochondrial fission and inhibits mitophagy activity. Mechanistically, mitochondrial fission/fusion and mitophagy activity interact reciprocally with each other and are both involved in postoperative dNCR. These mitochondrial events after surgical stress may provide novel targets and modalities for therapeutic intervention in postoperative dNCR.

## Graphical abstract

Mitochondrial fission and mitophagy reciprocal interactions in postoperative dNCR. Laparotomy increases Drp1‐induced mitochondria fission and inhibits mitophagy in the aged hippocampus, thus causing mitochondrial dysfunction and postoperative dNCR. Mitochondrial hyperfission and mitophagy inhibition coexist and interact with each other, which is expected to become a potential intervention target for dNCR after surgery.

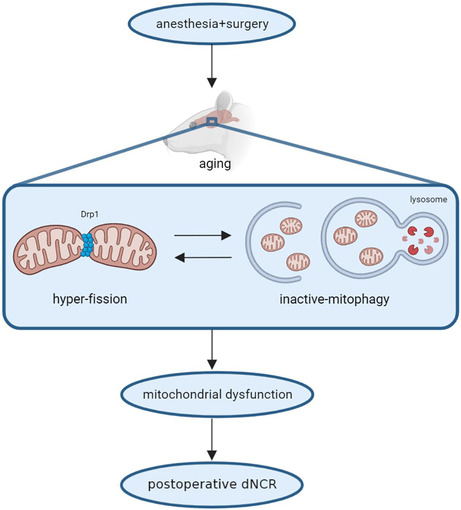

## 1 INTRODUCTION

Delayed neurocognitive recovery (dNCR) after surgery refers to the cognitive impairment of patients within 30 days from surgery.^1^ The cognitive impairment refers to a severe decline in memory, attention, and social interaction, as well as personality changes; these impairments seriously affect the quality of life, prolong hospital stays, and bring severe financial burden to patients and their families.^2^ However, the mechanism of dNCR in geriatric patients remains unclear. Effective methods for preventing and treating dNCR are therefore imperative.

Increasing evidence suggests that mitochondrial damage is strongly involved in the mechanism of postoperative dNCR. Long duration of anesthesia exposure or harmful stimulation caused by surgery may damage mitochondrial function, affect its supply of energy to neurons, and cause cytotoxicity, thus leading to the occurrence of dNCR.^3^, ^4^ Studies have reported that aged rats display memory impairment accompanied by mitochondrial dysfunction and oxidative damage after tibial fracture.^5^ Furthermore, Wang et al.^6^ revealed that laparotomy may induce oxidative stress, mitochondrial dysfunction, and autophagy dysregulation, resulting in cognitive impairment. In addition, the accumulation of lipid droplets in the hippocampus accelerates damage to mitochondria after surgery.^7^ Our previous study demonstrated that anesthesia and surgical trauma increase mitochondrial α‐synuclein accumulation, disrupt mitochondrial homeostasis, and promote mitochondria‐dependent apoptosis.^8^ Together, these studies indicate that mitochondria, which are the main target organelles of oxidative damage and guarantee the survival and function of neurons, may play an essential role in the progress of postoperative dNCR.^9^, ^10^

Normally, mitochondria maintain internal homeostasis through a continuous process of fission/fusion, mitophagy, mitochondrial transport, and mitochondrial biogenesis.^11^ During these processes, mitochondria remove neuronal “junk” by adjusting their morphology in sequential fusion/fission rounds and then using mitophagy to maintain a healthy status. They exchange mitochondrial DNA (mtDNA) and complete mitochondrial metabolites through fusion.^12^ Damaged mitochondria obtain normal components to be repaired through fusion, while irreparably damaged mitochondria are removed by fission and eliminated by mitophagy to ensure the optimal functioning of mitochondria.^13^ An abnormal dynamic balance of mitochondrial fission/fusion or mitophagy may affect mitochondrial function and cause several neurodegenerative diseases.^14^

Given the important roles of mitochondrial fission/fusion and mitophagy in the maintenance of cellular homeostasis, their roles in dNCR have attracted great attention. Studies have shown that exploratory laparotomy under 1.4% isoflurane for 2 h leads to excessive mitochondrial fission in neurons of the hippocampus and prefrontal cortex in aged mice.^15^ Yang et al.^16^ also reported that laparotomy under isoflurane increases dynamin‐related protein 1 (Drp1)‐regulated mitochondrial fragmentation in aged mice. Nevertheless, these morphological changes are only one part of the mitochondrial adaptive process. It remains unclear how mitophagy activity changes as mitochondrial morphology processes change. Simple anesthesia with 5% sevoflurane significantly inhibits mitophagy in aged mice.^17^ However, changes in mitochondrial fusion/fission and mitophagy, and how they regulate each other, in neurons after surgery in aged rats have not yet been reported.

In the present study, we used classic postoperative dNCR animal and cell models to observe the reciprocal interactions between postoperative mitochondrial morphology and mitophagy in hippocampal neurons in aged rats. Mitochondrial division inhibitor 1 (Mdivi‐1) was used to intervene in the excessive mitochondrial fission of neurons; rapamycin was used to activate mitophagy. Our findings provide new concepts and possible intervention targets for improving the postoperative outcomes of geriatric patients, as well as for the prevention and treatment of dNCR.

## 2 MATERIALS AND METHODS

### 2.1 Animals

Twenty‐month‐old male specific‐pathogen‐free Sprague Dawley rats weighing 500–600 g were purchased from Keyu Animal Breeding Center in Beijing, China. Animal experiments were conducted as per the regulations of The Laboratory Animal Welfare Ethics Branch of the Bioethics Committee of Peking University (No. LA2021433). All rats were kept in a laboratory in the Animal Science Department of Peking University Health Science Center with two rats per specific‐pathogen‐free cage under the following conditions: standard feeding, temperature 20–25°C, ambient humidity 40%–50%, 12‐h/12‐h light/dark cycle, adequate water, and a regularly cleaned cage and water bottle. Rats were allowed to adapt to the environment for at least 1 week before the experiment.

### 2.2 Anesthesia and surgical procedures

Aged rats were randomly divided into above groups by weight: (1) control group (Control), rats received no intervention; (2) anesthesia group (Sevoflurane), rats were anesthetized using 2.5% sevoflurane for 30 min; (3) anesthesia and surgery group (Surgery), exploratory laparotomy procedures were performed under sevoflurane anesthesia to establish the dNCR model, as described in other studies.^18^, ^19^ Each rat was placed into a sevoflurane anesthesia machine and received 2.5% sevoflurane for 5 min. Next, the rat was fixed on the operating table while maintaining effective ventilation; the sevoflurane concentration was maintained at 2.5%. The abdominal skin was revealed, disinfected with iodine, and wiped twice with 75% alcohol to remove the iodine. A 5‐cm incision was then made to expose the intestinal tissue, and the liver, spleen, and intestine tissue were probed. Subsequently, 5 cm of the small intestine was removed, rubbed between the thumb and forefinger for about 30 s, and returned to the abdominal cavity. The skin tissue was sutured layer by layer with surgical thread, the incision was disinfected with iodophor, and bupivacaine analgesic gel was applied. The rat was then placed on a warm blanket to wake up naturally. The operation duration was controlled at about 30 min; (4) surgery plus Mdivi‐1 group (Surgery+Mdivi‐1), rats were intraperitoneally injected with DMSO dissolved Mdivi‐1 at a dose of 20 mg/kg 4 h before anesthesia; (5) Mdivi‐1 alone group (Mdivi‐1), the injection procedure was consistent with that of Surgery+Mdivi‐1 group.

### 2.3 Drug administration

Rats were intraperitoneally injected with dimethyl sulfoxide‐dissolved Mdivi‐1 (20 mg/kg; Sigma) 4 h before surgery.^20^, ^21^ Cells were treated with dimethyl sulfoxide‐dissolved rapamycin (100 nM; Sigma) in the presence of LPS (1 μg/mL) for 24 h.^22^, ^23^

### 2.4 Morris water maze (MWM) test

Mild but significant cognitive deficits can be detected by the MWM test or conditioned fear test within 1–2 weeks after surgery in aged rodents.^24^ We therefore used the MWM (Sunny Instruments Co. Ltd.) test to measure hippocampus‐dependent spatial learning and memory. A circular black pool was divided into four even quadrants and shielded by light‐blocking curtains to prevent interference from reference objects or experimenters. Stickers of different shapes were affixed to the inner side of the light‐blocking curtain in each quadrant to facilitate learning of the environment in the aged rats. In the pool, a platform was placed in the target quadrant; the water level was 0.5–1 cm higher than the platform. An appropriate amount of black ink was poured into the water and mixed evenly to ensure that the rats could not see the position of the platform, and the water temperature was maintained at 35°C.

For five consecutive days before surgery, rats were placed into the pool in each of the different quadrants as training. The swimming trajectory, speed, and time to find the platform were recorded. If a rat was unable to find the platform in 2 min, it was placed on the platform to familiarize itself with the environment for at least 30 s.

The rats did not swim on the day of surgery but swam for 2 days after surgery. The platform in the target quadrant was removed on postoperative day 3. The number of times each rat crossed the space where the platform had been was recorded along with the time spent in the target quadrant. Hippocampal tissue was removed on postoperative day 3.

### 2.5 Transmission electron microscopy (TEM) imaging and mitochondrial assessment

On postoperative day 3, the hearts of the aged rats were exposed and perfusion fluid was injected into the left ventricle. At the same time, the right atrial appendages were cut, perfused with phosphate‐buffered saline (PBS) until the blood was completely replaced, and then injected with 25% glutaraldehyde and 0.2 MPBS mixed fixative solution using a three‐way tube. Rats were perfused for about 30 min at a rate of 1 drop/s; the perfusion volume was 200 mL. Hippocampal tissue was then removed, cut into 1 mm^3^ pieces, and fixed in 2.5% glutaraldehyde for 24 h followed by 1% osmic acid for 2 h. Next, the tissue was dehydrated using acetone and embedded in epoxy. Ultrathin slices (70–80 nm) were sectioned on an ultramicrotome. A JEOL JEM‐1400 was then used to observe the ultrastructure of hippocampal mitochondria at 1000× magnification. The mitochondrial morphology data were analyzed for major/minor axes using Image J software.^25^ Mitochondria form factor was measured from three rats and 50 cells per group; the mitochondrial aspect ratio was measured from three rats and 10 cells per group.^26^ The form factor was calculated as the perimeter^2^/(4π × area), while the aspect ratio was calculated as the mitochondrial maximum Feret diameter/minimum Feret diameter.^25^

### 2.6 Western blotting analysis

The hippocampus or cells were lysed in radioimmunoprecipitation assay buffer with protease and phosphatase inhibitors and homogenized for 2 min. Protein concentrations were then measured using a Bicinchoninic Acid (BCA) Protein Assay Kit (Beyotime, P0010). The loading volume was calculated according to a protein content per well of 40 μg; the experimental protocol was consistent with that of a previous study.^27^ After blocking with 5% Albumin Bovine V (Solarbio, A8020) in Tris‐buffered saline containing 0.1% Tween 20 for 1 h at room temperature, membranes were incubated overnight at 4°C with the following primary antibodies: anti‐Drp1, anti‐Fis1, anti‐OPA1, anti‐Mfn1, anti‐Mfn2 (Santa Cruz, CA, USA), anti‐p‐Drp1 (Ser637), anti‐p‐Drp1 (Ser616), anti‐cytochrome oxidase subunit IV (COXIV; Cell Signaling Technology), anti‐sequestosome 1, (p62; Cell Signaling Technology), anti‐microtubule‐associated protein 1 light chain 3β, (LC3B, Cell Signaling Technology and Abcam), and anti‐superoxide dismutase 2, (SOD2, Proteintech). Subsequent incubation was performed using goat anti‐rabbit/mouse secondary antibodies for 1 h at room temperature. The membranes were then imaged via enhanced chemiluminescence using an Odyssey infrared imaging system (LI‐COR Biosciences).

### 2.7 Immunofluorescence staining

The frozen sections were warmed to room temperature and rinsed with PBS on a shaker for 15 min. A circle was then drawn around the tissue section with an immunohistochemical pen, and blocking solution was added for 1 h at room temperature. Sections were incubated overnight at 4°C in a working solution containing primary antibody against LC3B and COXIV proteins; a negative control was included. The sections then underwent three 15‐min washes in PBS on a shaker before they were placed in a wet box for 1 h in the dark at room temperature with two different kinds of secondary antibody working solutions. After three washes with PBS on a shaker, 4′,6‐diamidino‐2‐phenylindole was added to counterstain the nuclei. An anti‐fluorescence quenching sealing reagent was used for coverslipping before sections were imaged with Leica microsystems.

### 2.8 Quantitative reverse transcription polymerase chain reaction (qRT‐PCR)

The messenger RNA (mRNA) expression levels of *Drp1*, *Fis1*, *Opa1*, *Mfn1*, *Mfn2*, and *Actb* (encoding β‐actin) were measured using qRT‐PCR. Total mRNA was isolated from the hippocampus using Trizol (Invitrogen) and quantified with a NanoDrop 2000c system (Thermo Fisher Scientific). The sequence details of individual pairs of primers are shown in Table 1; the experimental protocol has been described previously.^28^ The PCR consisted of 40 cycles of 5 s at 95°C and 15 s at 60°C. The relative mRNA levels were measured using the 2^−Δ(Δ CT)^ method and normalized to *Actb* levels.

**TABLE 1:** Sequences of primers used for the quantitative reverse transcription polymerase chain reaction (qRT‐PCR) analysis.

mRNA	Primer	Sequence (5′–3′)
*drp1*	Sense Antisense	GAGAACTACCTTCCGCTGTATCGC CACCATCTCCAATTCCACCACCTG
*fis1*	Sense Antisense	GAATACGCCTGGTGCCTGGTTC GAAGACATAATCCCGCTGCTCCTC
*opa1*	Sense Antisense	ATGCTCGCTATCACTGCCAACAC CCTTCTTCTCGCCGTCTTCAGC
*mfn1*	Sense Antisense	CGTGGCAGCAGCAGAGAAGAG CCTCCTCCGTGACCTCCTTGATC
*mfn2*	Sense Antisense	TCCACAGCCATTGCCAGTTCAC CCGCACAGACACAGGAAGAAGG
*actb*	Sense Antisense	CGTTGACATCCGTAAAGACCTC TAGGAGCCAGGGCAGTAATCT

### 2.9 Tissue reactive oxygen species (ROS) detection

Hippocampal tissue (30 mg) was taken from aged rats on ice, rapidly homogenized, lysed, and centrifuged at 12,000 ***g*** for 10 min to obtain the supernatant. Dichlorodihydrofluorescein diacetate was diluted at a concentration of 1:1000 and incubated at 37°C for 20 min. Fluorescence intensity was detected with an excitation wavelength of 488 nm and an emission wavelength of 525 nm.

### 2.10 Tissue mitochondria isolation

Hundred milligram fresh hippocampus tissue was cut into 0.5 cm^2^ pieces and homogenized with 1.5 mL Mito Solution (C0010, Pulilai) on ice. Homogenates were centrifuged at 800 ***g*** for 5 min. Then, collect the supernatant and centrifuge at 800 ***g*** for 5 min. Collect the supernatant and centrifuge at 10,000 ***g*** for 10 min. Add 0.2 mL Mito Solution to suspend mitochondrial precipitation and centrifuge at 12,000 ***g*** for 10 min. Discard the supernatant and resuspend mitochondria with a storage buffer.

### 2.11 Cell adenovirus transfection

Adenovirus (ADM‐CMV‐MCherry‐EGFP‐LC3B) was purchased from Shandong Weizhen Biotechnology with a titer of 4.2 × 10^10^ plaque‐forming units/mL.

HT22 cells were plated in a 24‐well plate the day before transfection, and the required amount of virus was calculated according to a multiplicity of infection of 5, 10, and 20. On the day of transfection, the virus was added to the prepared cell medium, and the cell medium (without penicillin/streptomycin) was changed after 4 h. After 8 h, the medium was replaced with culture medium containing 1 μg/mL lipopolysaccharide (LPS), which was left for 24 h. Cell staining was observed under a fluorescence microscope.

### 2.12 Small interfering RNA (siRNA) transfection

The siDrp1 was purchased from Beijing Pulizhicheng Biotechnology. The sequence of mouse *Drp1*‐siRNA was as follows:

Forward: 5′‐GCAGAACUCUAGCUGUAAUTT‐3′.

Reverse: 5′‐AUUACAGCUAGAGUUCUGCTT‐3′.

The HT22 cells were placed in six‐well plates and transfected when they grew to 30%–50% confluence. Four sterile Eppendorf tubes were labeled negative control (NC), Drp1, lip2000‐NC, and lip2000‐Drp1, respectively. First, 200 μL Opti‐MEM was added to each tube. Next, 4 μL NC was added to the NC tube, 4 μL Drp1 was added to the Drp1 tube, and 4 μL Lipofectamine 2000 was added to the other two tubes. The tubes were then left for 5 min. Subsequently, the reagent in the NC tube was removed and added to the lip2000‐NC tube, and the reagent in the Drp1 tube was added to the lip2000‐Drp1 tube; the tubes were left for 20 min. The medium in the six‐well plate was changed to 1.6 mL Opti‐MEM per well, and 400 μL of the mixed solution in each group was added dropwise into the six‐well plate. The plate was then incubated for 4 h in a cell incubator. Finally, the medium was replaced with cell medium without penicillin/streptomycin for subsequent experiments.

### 2.13 Mitochondrial ROS (mtROS) detection

Cells were cultured in 24‐well plates, and 13 μL dimethyl sulfoxide was added to 50 μg MitoSOX (M36008; Thermo Fisher) and diluted with medium to make a working concentration of 5 μM. Next, 1 mL MitoSOX was added to each well, and the cells were incubated at 37°C for 10 min in the dark. After being washed three times with PBS, they were observed under a microscope. For isolated hippocampal mitochondria, 10 μg mitochondria were mixed with 100 μL MitoSOX working solution in a 96‐well plate and were observed under a fluorescent enzyme reader.

### 2.14 Enhanced adenosine triphosphate (ATP) detection

Cells were cultured in a six‐well plate and the medium was removed. Next, 200 μL lysate was added to each well, mixed with a pipette gun, and centrifuged at 12,000 ***g*** at 4°C for 5 min. The ATP standards (S0027; Beyotime) were then diluted to prepare ATP working solutions; 100 μL working solution was added to each well and left at room temperature for 5 min to reduce the background. Finally, 20 μL of sample or standard was added to each well and detected under a luminescence microplate reader.

### 2.15 Determination of mitochondrial membrane potential (MMP)

Cells were incubated in a six‐well plate, culture medium was removed, and the cells were incubated at 37°C for 20 min in 1 mL JC‐1 (C2006; Beyotime) staining working solution. The JC‐1 staining buffer was prepared at a ratio of 4 mL distilled water to 1 mL buffer (5×). Following incubation, the supernatant was removed and cells were washed twice with JC‐1 buffer. Finally, 2 mL cell medium was added and the cells were observed under a laser confocal microscope. For isolated hippocampal mitochondria, 10 μg mitochondria were mixed with 90 μL JC‐1 staining working solution in a 96‐well plate and were observed under a fluorescent enzyme reader.

### 2.16 Mito‐tracker staining

The mito‐tracker red dye (C1035; Beyotime) was diluted at 1:1000 in cell medium and the cells were incubated at 37°C for 30 min. The cells were observed under the fluorescence microscope.

### 2.17 Statistical analysis

The statistical analysis was processed based on the IBM SPSS statistics 25.0 (SPSS). Shapiro–Wilk test was used to evaluate whether the data conform to normal distribution, and we found the data were normally distributed. The two‐sample *t*‐test and a one‐way analysis of variance (ANOVA) were used for normally distributed variables. GraphPad Prism 8.0 was used to analyze the data. The results of at least three independent experiments were selected for statistical analysis. Measurement data are expressed as the mean ± standard error of the mean, and *p* < 0.05 was considered significant.

## 3 RESULTS

### 3.1 Surgical stress‐induced spatial learning and memory impairments in aged rats

**FIGURE 1:**
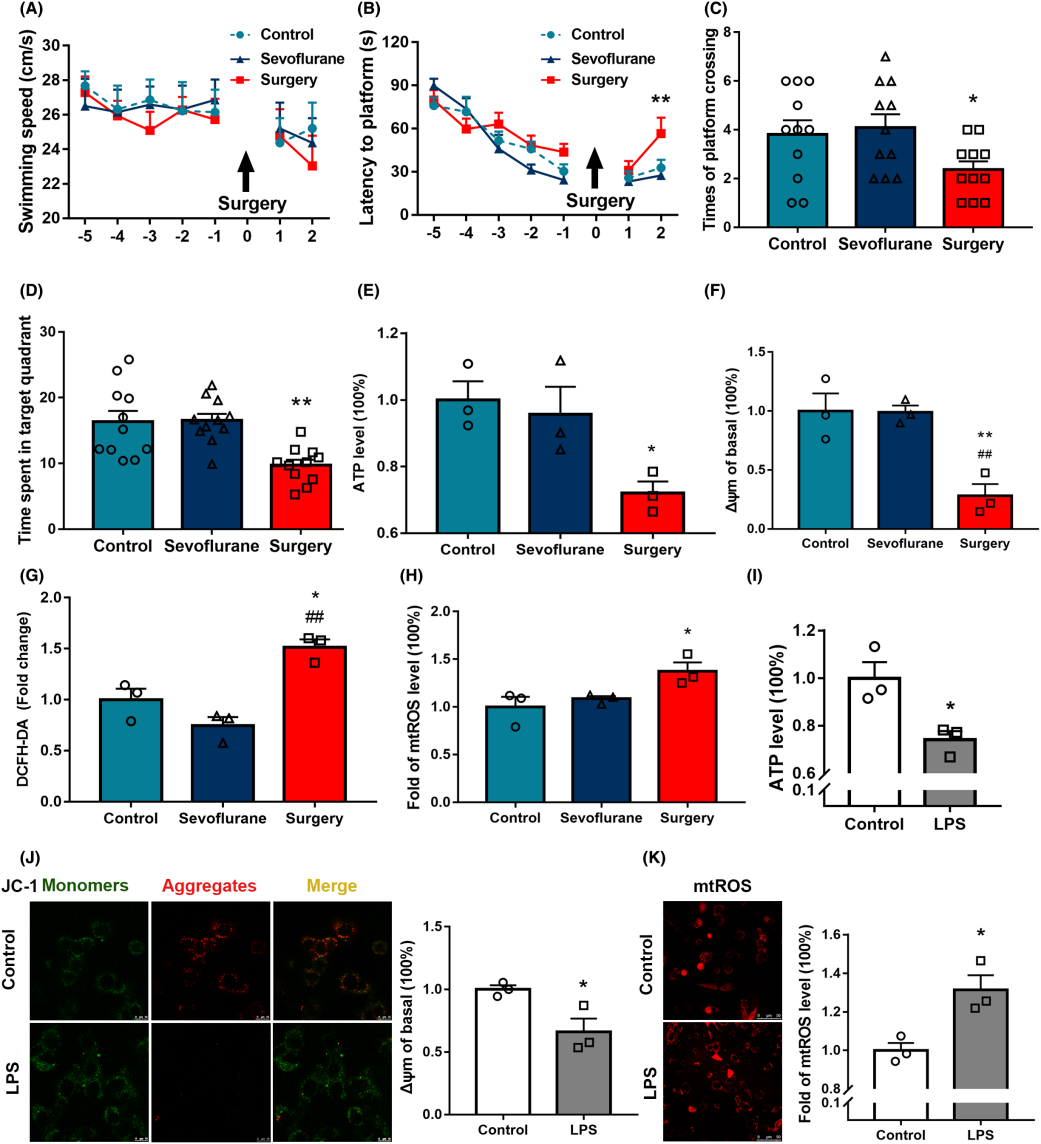
Surgery‐induced spatial learning and memory deficits parallel mitochondrial dysfunction in vivo and in vitro. (A) Swimming speed of aged rats in the MWM test. (B) Latency to the platform. (C) Numbers of platform crossings. (D) Time spent in the target quadrant (*n* = 11). (E) ATP levels in the hippocampus of aged rats after exploratory laparotomy under sevoflurane anesthesia. (F) MMP levels of mitochondria in the hippocampus of aged rats. (G) ROS levels in the hippocampus of aged rats. (H) mtROS levels of mitochondria in the hippocampus of aged rats. (*n* = 3). Control vs. Surgery, *p < 0.05, **p < 0.01; Sevoflurane vs. Surgery, #*p* < 0.05, ##*p* < 0.01. Mitochondrial function of HT22 cells was detected after 24‐h induction with 1 μg/mL LPS. (I) Changes in ATP production. (J) Mitochondrial membrane potential was detected using JC‐1, scale bar = 10 μm. (K) Changes in mitochondrial ROS, scale bar = 50 μm (*n* = 3). Data are expressed as the mean ± standard error. Control vs. LPS, *p < 0.05.

The MWM was used to assess hippocampus‐dependent spatial learning and memory of aged rats after laparotomy under sevoflurane anesthesia. There were no significant differences in swimming speed among the Control, Sevoflurane, and Surgery groups (Figure 1A). On postoperative day 2, the latency to the platform in the Surgery group was significantly increased than the Control group (Figure 1B); there was no significant difference between the Control and Sevoflurane groups (Figure 1B). On postoperative day 3, the number of platform crossings and time spent in the target quadrant were significantly decreased in the Surgery group compared with the Control group, while there was no significant difference between the Control and Sevoflurane groups (Figure 1C,D). These results indicate that exploratory laparotomy under sevoflurane anesthesia induces spatial learning and memory decline in aged rats.

### 3.2 Roles of mitochondrial dysfunction in postoperative dNCR

To evaluate the mitochondrial oxidative stress response in the hippocampus of aged rats with dNCR, hippocampal ATP, MMP, and mtROS levels were detected. Surgery decreased ATP and MMP levels compared with the Control (*p* < 0.05, Figure 1E,F). The hippocampal ROS and mtROS were significantly increased after surgery (*p <* 0.05, Figure 1G,H). These results suggest that mitochondrial dysfunction in the hippocampus of aged rats after surgery may be closely related to the occurrence of dNCR.

To further explore the role of mitochondrial dysfunction in LPS‐induced HT22 neurons, we treated the cells with 1 μg/mL LPS for 24 h and then detected mtROS, MMP, and ATP levels. Both ATP production and MMP were decreased (Figure 1I,J) and mtROS levels were increased (Figure 1K) after the LPS induction of HT22 neurons.

### 3.3 Increased mitochondrial fission of hippocampal neurons in aged rats after surgery

**FIGURE 2:**
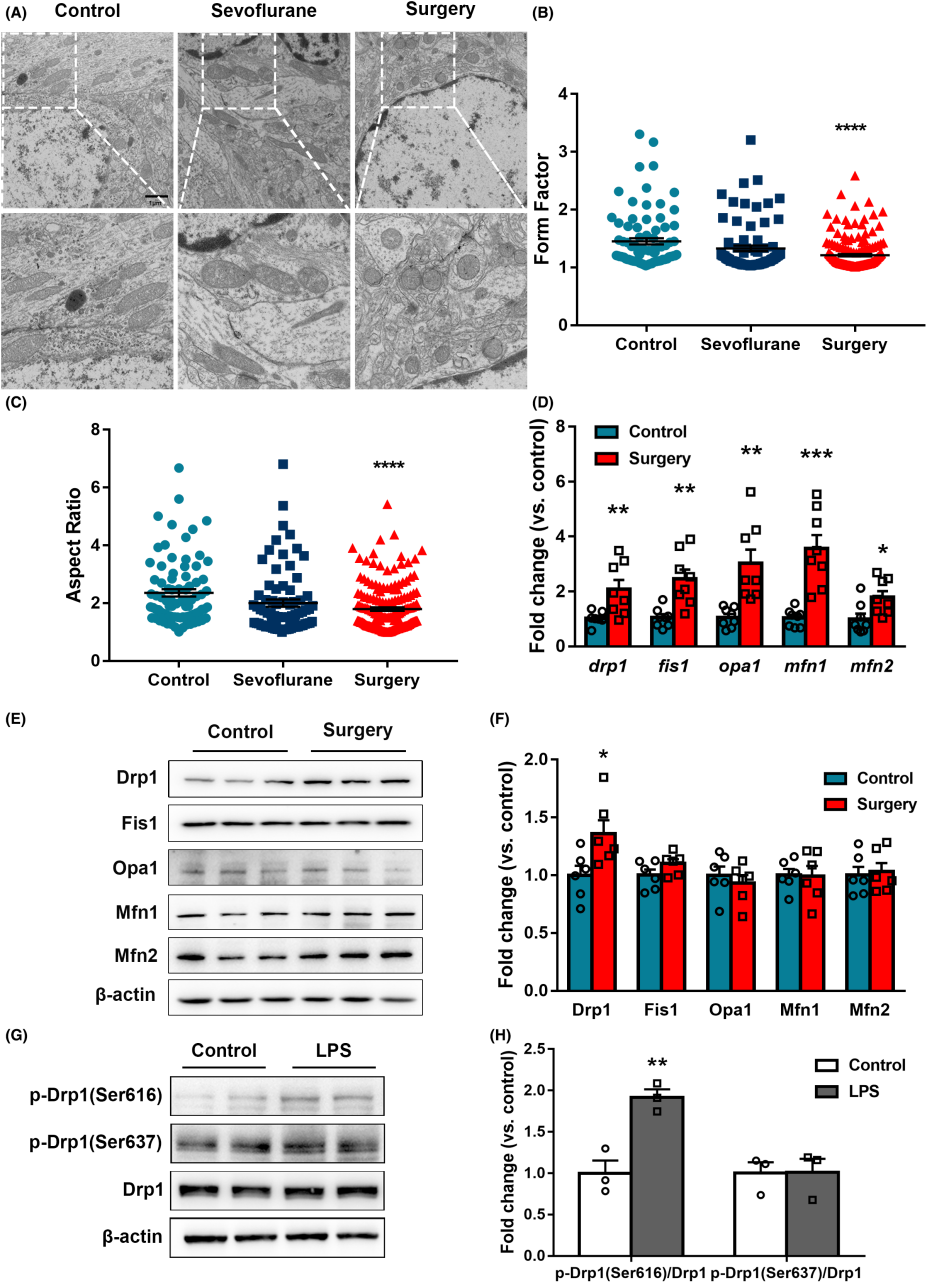
Increased mitochondrial fission in the aged hippocampus after surgery and in LPS‐induced neurons. (A) Mitochondrial morphology was observed using TEM on postoperative day 3, scale bar = 1 μm. (B) Mitochondrial form factor (*n* = 50 cells/group). (C) Mitochondrial aspect ratio (*n* = 10 cells/group, Control: *n* = 82 mitochondria, Sevoflurane: *n* = 76 mitochondria, Surgery: *n* = 184 mitochondria). (D) mRNA changes of mitochondrial fission and fusion proteins in the hippocampus. (E, F) Mitochondrial fission and fusion proteins in the hippocampus (*n* = 6). (G, H) Drp1 and its phosphorylation at Ser616 and Ser637 in HT22 cells induced by 1 μg/mL LPS for 24 h. Data are expressed as the mean ± standard error. Control vs. Surgery, ***p < 0.001, ****p < 0.0001; Control vs. LPS, *p < 0.05, **p < 0.01.

Mitochondrial morphology is an essential early‐phase change in mitochondrial function. TEM was used to observe the aspect ratios and form factors of mitochondria around the nuclei of hippocampal neurons in aged rats after surgery. Mitochondrial morphology in the Control and Sevoflurane groups was mostly elongated and club‐shaped, whereas mitochondria in the Surgery group were mostly small and round (Figure 2A). The aspect ratio and form factor of mitochondria in the Surgery group were decreased compared with the Control group (Figure 2B,C); there were no significant differences between the Control and Sevoflurane groups.

Next, changes in mitochondrial fission and fusion regulatory proteins were explored at the gene transcription level. The mRNA expression levels of *Drp1*, *Fis1, Opa1, Mfn1*, and *Mfn2* in hippocampal tissues were detected using qRT‐PCR (Figure 2D). The transcription levels of all five fission and fusion regulatory genes were increased to varying degrees in the Surgery group compared with controls (all *p* < 0.05). Then, we detected the expression level of fusion and fission proteins. The results suggested that only Drp1 increased significantly among these proteins after surgery (Figure 2E,F).

To further explore the specific mechanism of increased mitochondrial fission, HT22 cells were stimulated with 1 μg/mL LPS for 24 h. The protein expression levels of Drp1 and its Ser616 and Ser637 phosphorylation were detected. Compared with the Control group, there were no significant differences in the phosphorylation levels of Drp1 or p‐Drp1 (Ser637) in the LPS‐treated cells (*p* < 0.05), whereas p‐Drp1 (Ser616) was significantly increased (*p* < 0.01, Figure 2G,H). These results indicate that p‐Drp1 (Ser616) promotes neuronal mitochondrial fission.

### 3.4 Surgery inhibits mitophagy in the hippocampal neurons of aged rats

**FIGURE 3:**
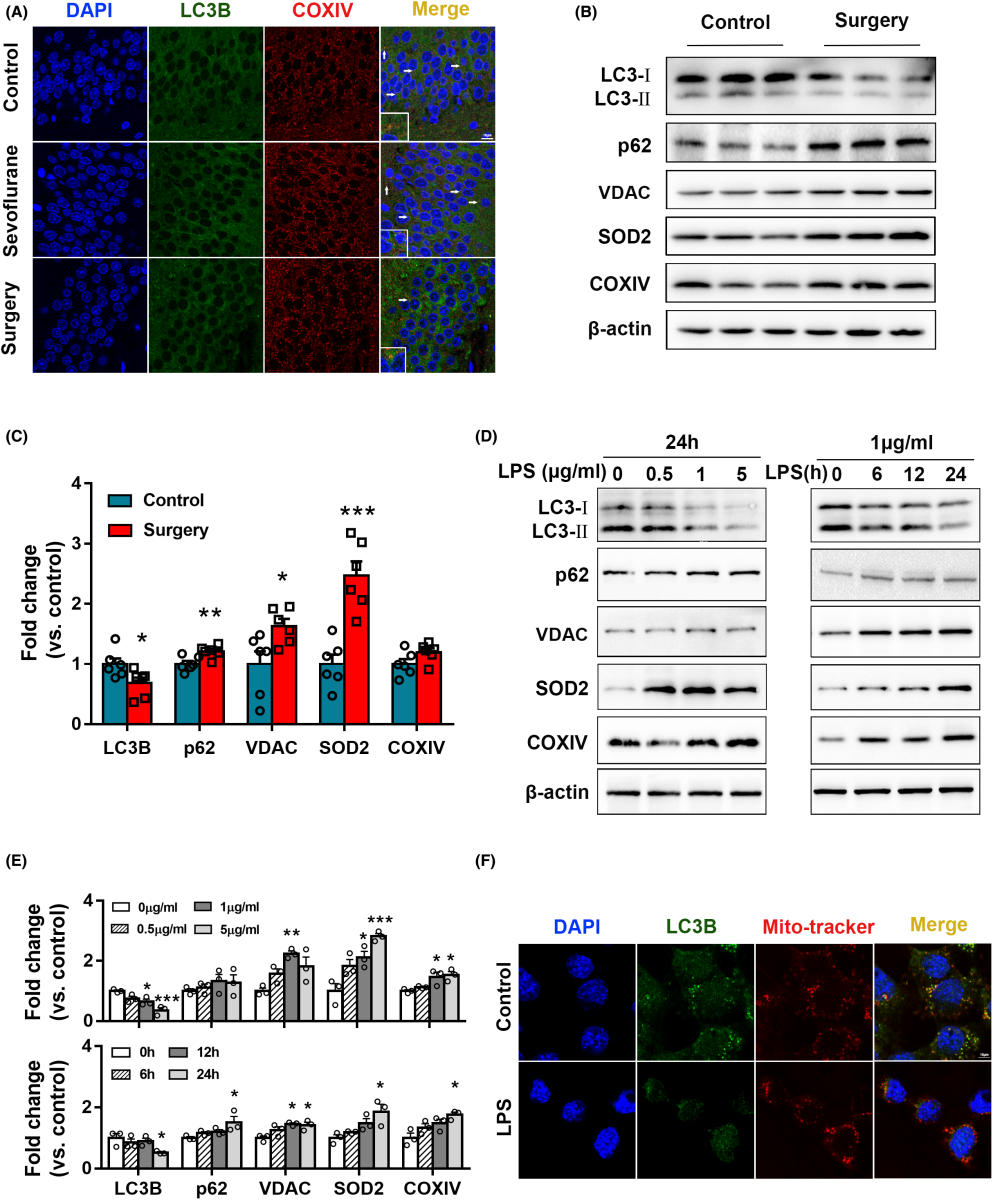
Inhibited mitophagy in the surgery‐challenged aged hippocampus and LPS‐induced neurons. (A) LC3B colocalized with COXIV in the CA1 region of the rat hippocampus, scale bar = 1 μm. (B, C) Levels of mitophagy‐related proteins of hippocampus were analyzed by western blotting (*n* = 6). (D, E) HT22 cells were treated with LPS at different concentrations and times, as indicated. Levels of mitophagy‐related proteins were analyzed by western blotting. 1 μg/mL vs. 0 μg/mL, *p < 0.05, **p < 0.01; 5 μg/mL vs. 0 μg/mL, *p < 0.05, ***p < 0.001; 24 h vs. 0 h, *p < 0.05. (F) Co‐localization of LC3B and Mito‐tracker in HT22 cells induced by 1 μg/mL LPS for 24 h, scale bar = 10 μm, *n* = 3.

Mitochondrial fission is closely related to mitophagy. To explore changes in mitophagy after surgery, immunofluorescence was used to detect the co‐localization of LC3B and COXIV in the hippocampal CA1 region. There were significantly fewer cells with co‐localization in the Surgery group than in the Control and Sevoflurane groups (Figure 3A); there was no significant difference between the Control and Sevoflurane groups. The expression of LC3B, p62, VDAC, SOD2, and COXIV was also detected (Figure 3B,C). Surgery decreased LC3‐II/LC3‐I level and increased p62, VDAC, SOD2, and COXIV. These results indicate that mitophagy is inhibited in the hippocampal CA1 region of aged rats on postoperative day 3.

To investigate the effect of LPS on mitophagy in HT22 cells, cells were induced by LPS at varying concentrations (0.5, 1, and 5 μg/mL) for 24 h, and at 1 μg/mL for different time periods (6, 12, and 24 h). The expression of LC3B, p62, VDAC, SOD2, and COXIV was then detected (Figure 3D,E). The expression of LC3‐II/LC3‐I decreased 24 h after 1 μg/mL LPS induction (*p* < 0.05), whereas VDAC, SOD2, and COXIV were significantly increased (all *p* < 0.05). Furthermore, LC3‐II/LC3‐I was significantly decreased after 5 μg/mL LPS induction for 24 h (*p* < 0.001), whereas SOD2 and COXIV were significantly increased (both *p* < 0.05). Considering that 24 h of 1 and 5 μg/mL LPS induction had similar inhibitory effects on mitophagy in HT22 neurons, 1 μg/mL LPS was selected for all subsequent experiments. The expression of LC3‐II/LC3‐I after 1 μg/mL LPS for 24 h was significantly decreased (*p* < 0.05), whereas the expression of p62, VDAC, SOD2, and COXIV was markedly increased (all *p* < 0.05) (Figure 3D,E). Thus, the inhibition of mitophagy was most effective in HT22 cells induced with 1 μg/mL LPS for 24 h.

The co‐localization of LC3B and Mito‐tracker was detected by immunofluorescence in LPS‐induced HT22 cells. The co‐localization of these two proteins was significantly reduced in the LPS group (Figure 3F), suggesting that mitophagy was inhibited.

### 3.5 Inhibition of mitochondrial fission promotes mitophagy and improves neurocognitive function after surgery

**FIGURE 4:**
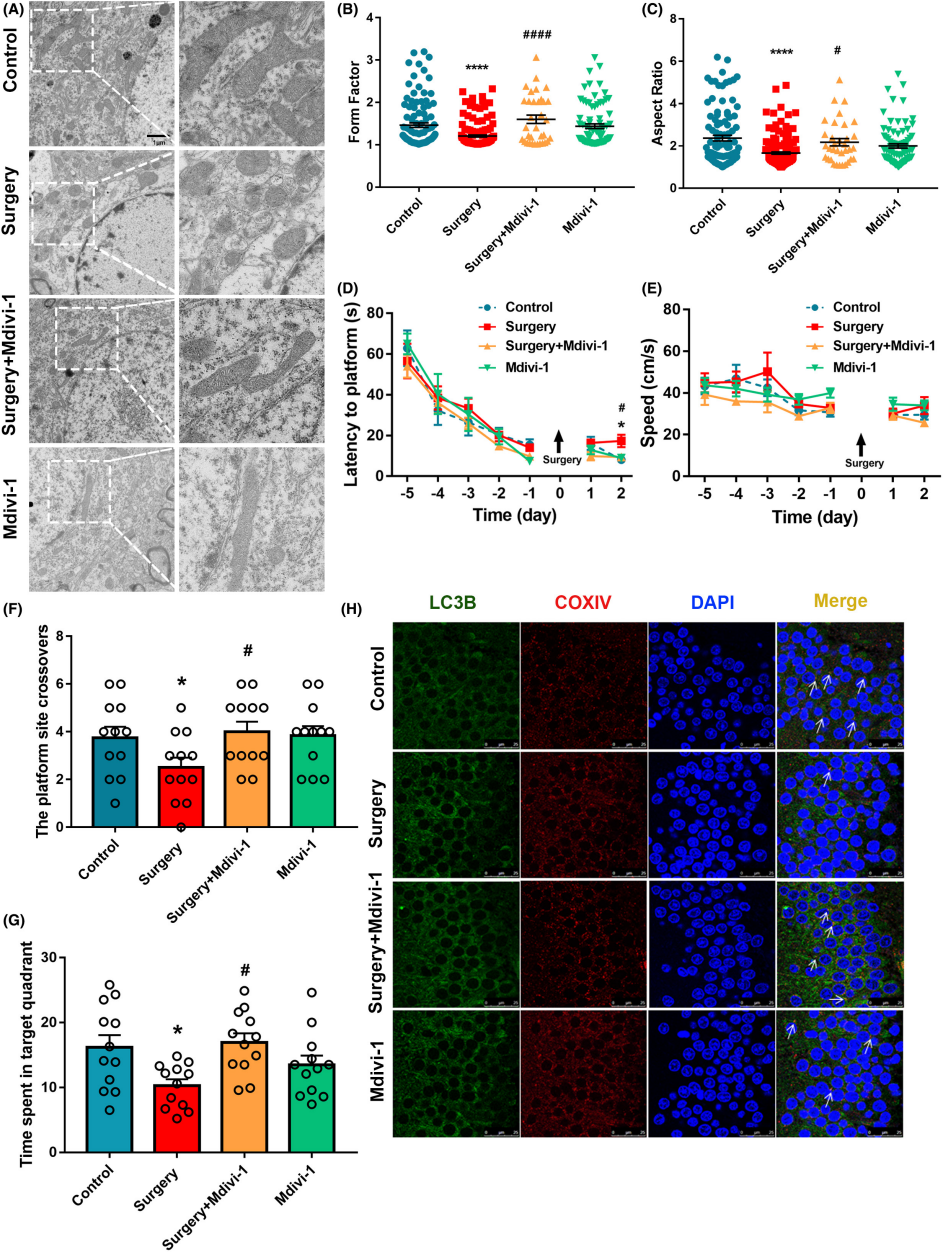
Mdivi‐1, a mitochondria division inhibitor, improves postoperative neurobehavioral function in aged rats. (A) Mitochondrial morphology was observed in the hippocampus of aged rats pretreated with Mdivi‐1 using TEM, scale bar = 1 μm. (B) Mitochondrial form factor (*n* = 50 cells/group). (C) Mitochondrial aspect ratio (*n* = 10 cells/group, Control: *n* = 104 mitochondria, Surgery: *n* = 157 mitochondria, Surgery+Mdivi‐1: *n* = 34 mitochondria, Mdivi‐1: *n* = 81 mitochondria). (D) MWM latency to the platform. (E) MWM swimming speed. (F) MWM number of platform crossings. (G) MWM time spent in the target quadrant. (H) Co‐localization of LC3B and COXIV in the hippocampal CA1 region, scale bar = 25 μm. Control vs. Surgery, *p < 0.05, **p < 0.01, ****p < 0.0001, Surgery+Mdivi‐1 vs. Surgery, #*p* < 0.05, ##*p* < 0.01, ####*p* < 0.0001, *n* = 3.

Mdivi‐1, a specific inhibitor of the mitochondrial fission protein Drp1, can effectively inhibit excessive mitochondrial fission in rat hippocampal neurons.^20^ Twenty‐month‐old rats were randomly divided into four groups (*n* = 12): the Control, Surgery, Surgery+Mdivi‐1, and Mdivi‐1 groups. Mitochondrial morphology in hippocampal neurons was observed by TEM on postoperative day 3, and mitochondrial aspect ratios and form factors were calculated. Compared with the Surgery group, mitochondrial morphology in the Surgery+Mdivi‐1 group was restored to an elongated shape (Figure 4A). The aspect ratio and form factor of mitochondria decreased after surgery (Figure 4B,C); this effect was effectively inhibited by Mdivi‐1.

In the MWM on postoperative day 2, latency to the platform was shorter in the Surgery+Mdivi‐1 group than in the Surgery group (*p* < 0.05) (Figure 4D); the number of platform crossings and time spent in the target quadrant were also increased on postoperative day 3 (Figure 4F,G). There were no significant differences in swimming speed among the four groups (Figure 4E). These findings suggest that Mdivi‐1 improves postoperative neurobehavioral abnormalities in aged rats by inhibiting mitochondrial fission.

To explore the effect of mitochondrial fission on mitophagy, LC3B and COXIV were detected in neurons in the hippocampal CA1 region after mitochondrial fission was inhibited by Mdivi‐1 (Figure 4H). There was significantly more co‐localization of LC3B and COXIV in the Surgery+Mdivi‐1 group than in the Surgery group, indicating that the inhibition of mitochondrial fission can increase mitophagy.

**FIGURE 5:**
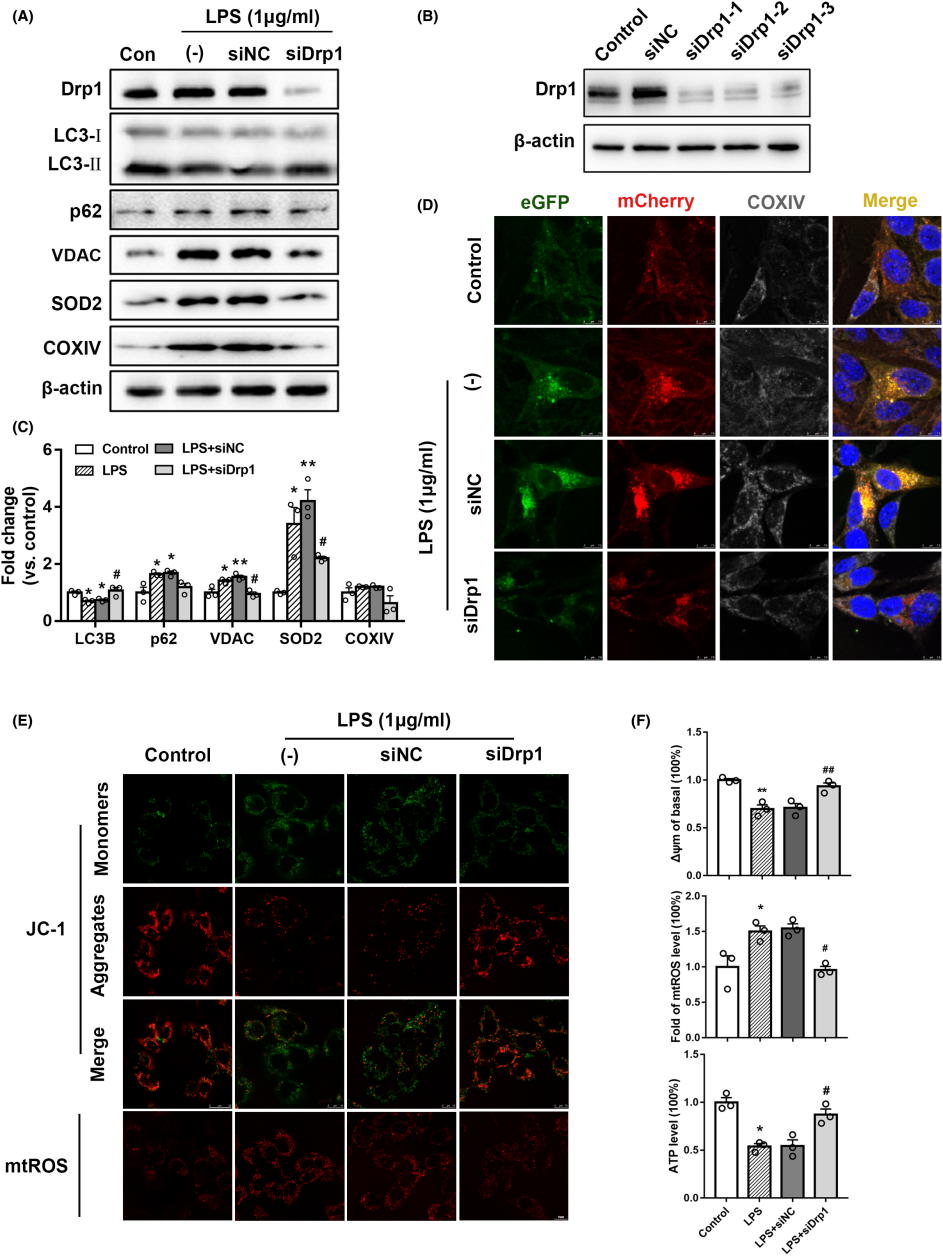
Drp1 knockdown improves mitophagy and mitochondrial dysfunction in vitro. (A–C) Western blotting detected the expression of mitophagy proteins in HT22 cells induced by 1 μg/mL LPS for 24 h after Drp1 knockdown. (D) eGFP‐mCherry‐LC3B AAV was used to detect changes in mitophagy flux induced by LPS in HT22 cells after Drp1 knockdown, scale bar = 7.5 μm. (E) MMP and mtROS levels in HT22 cells induced by LPS after Drp1 knockdown, scale bar = 10 μm. (F) Statistical analyses of MMP, mtROS, and ATP production, Control vs. LPS, *p < 0.05, **p < 0.01, LPS vs.LPS + siDrp1, #*p* < 0.05, ##*p* < 0.01, *n* = 3.

To further verify the effect of mitochondrial fission on mitophagy, siDrp1 was transfected into cells for 24 h to detect its effect. Western blotting revealed that all three siRNA were able to effectively knock down Drp1 (Figure 5B). Twenty‐four hours after the transfection of HT22 with siDrp1, the cells were induced with 1 μg/mL LPS for 24 h. This led to increased LC3‐II/LC3‐I expression and decreased VDAC and SOD2 expression (*p* < 0.05, Figure 5A,C), suggesting that the knockdown of Drp1 may improve mitophagy.

Next, eGFP‐mCherry‐LC3B AAV was used to detect changes in autophagy flow. When autophagosomes enter lysosomes, the acidic environment quenches the green fluorescence and only red is visible; when autophagosomes are inhibited, green fluorescence is not quenched and autophagosomes are observed as yellow. siDrp1 was first transfected into HT22 cells, and eGFP‐mCherry‐LC3B AAV was transfected 4 h later. The normal medium was replaced 4 h later. Cells were induced with 1 μg/mL LPS for 24 h, and COXIV immunofluorescence staining was then performed. The changes in autophagy flow and mitochondrial content were observed under confocal microscopy. Compared with the Control group, autophagy flow was inhibited with increased mitochondrial content in the LPS group. Furthermore, autophagy flow was restored and mitochondrial content was decreased in the LPS + siDrp1 group compared with the LPS group (Figure 5D). These results again suggest that Drp1 knockdown can increase mitophagy.

To explore the effect of mitochondrial fission inhibition on mitochondrial function, HT22 cells were infected with 1 μg/mL LPS for 24 h after transfection with siDrp1, and MMP, mtROS, and ATP production levels were detected. Compared with the LPS group, the MMP of the LPS + siDrp1 group recovered (*p* < 0.01), the mtROS level decreased (*p* < 0.05), and ATP production increased (*p* < 0.05, Figure 5E,F). These results suggest that Drp1 knockdown can improve mitochondrial function.

### 3.6 Activation of mitophagy alleviates excessive mitochondrial fission of HT22 cells and improves mitochondrial function

**FIGURE 6:**
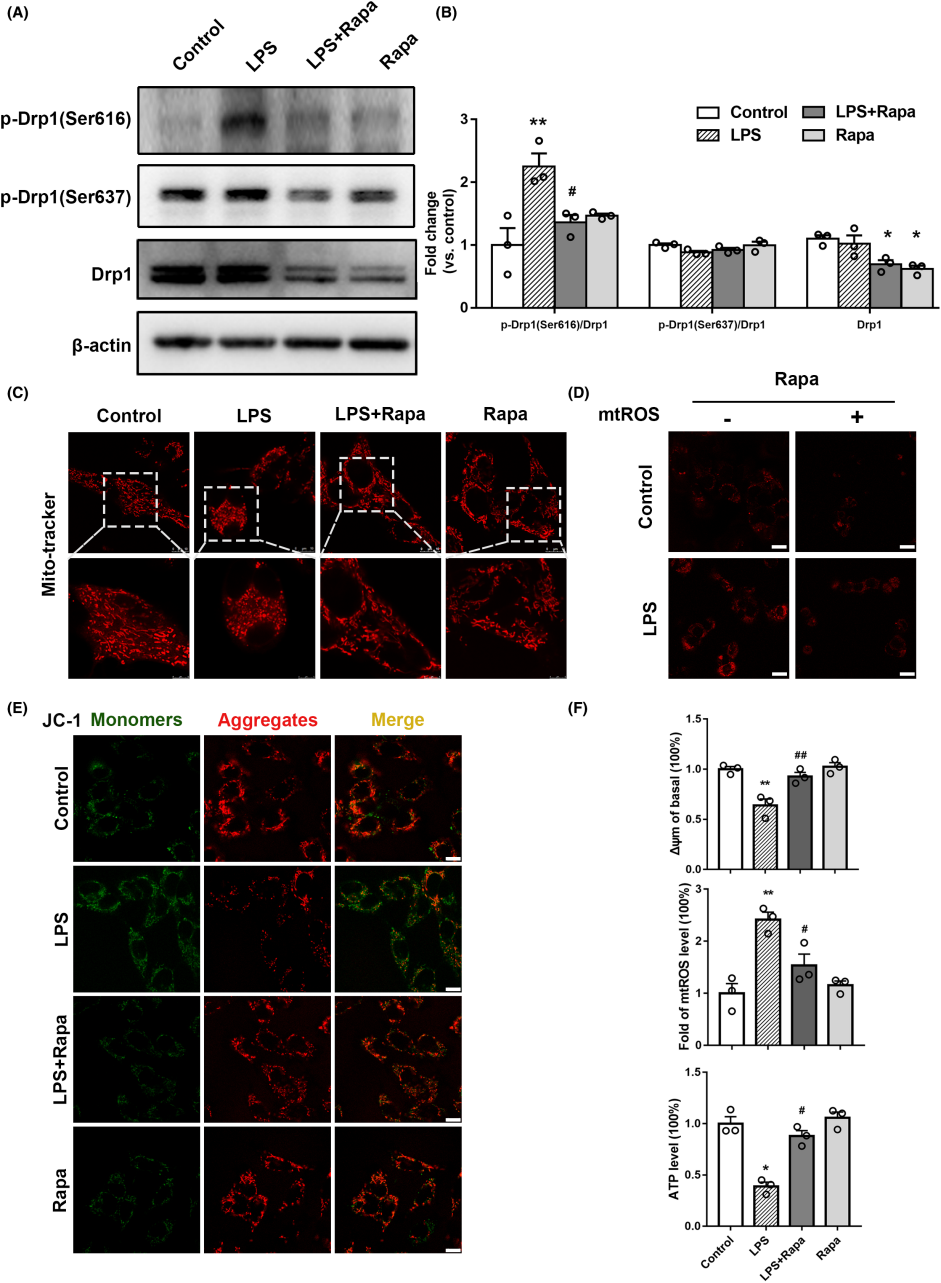
Activation of mitophagy by rapamycin attenuates LPS‐induced mitochondrial fission and improves mitochondrial function in vitro. (A, B) Effect of rapamycin on LPS‐induced Drp1, p‐Drp1(Ser616), and p‐Drp1(Ser637) expression in HT22 cells. (C) Effect of rapamycin on mitochondrial morphology of HT22 cells induced by LPS, scale bar = 10 μm. (D) Effect of rapamycin on mtROS levels in HT22 cells induced by LPS. (E) Effect of rapamycin on MMP levels in HT22 cells induced by LPS; scale bar = 10 μm. (F) Statistical analyses of MMP, mtROS, and ATP production level. Control vs. LPS, *p < 0.05, **p < 0.01; LPS vs. LPS + Rapa, #*p* < 0.05, ##*p* < 0.01; *n* = 3.

To investigate whether mitophagy affects mitochondrial fission, HT22 cells were induced with 1 μg/mL LPS and 100 nM rapamycin, which is an autophagy agonist. Next, Drp1 expression was detected. There was no significant difference in Drp1 expression between the Control and LPS groups, whereas Drp1 expression was decreased in the LPS + Rapa and Rapa groups (both *p* < 0.05, Figure 6A,B). Besides, the p‐Drp1(Ser616) level was decreased in the LPS + Rapa group when compared with the LPS group (*p* < 0.05), while the phosphorylation of DRP1 at Ser637 was comparable (Figure 6A,B). These findings suggest that promoting mitophagy effectively reduces Drp1 expression and phosphorylation.

To further explore the effect of promoting mitophagy on mitochondrial morphology, cells were administered LPS and rapamycin before the mitochondria were labeled with a red Mito‐tracker probe. Mitochondrial morphology in the four groups of cells was then observed under confocal microscopy. Compared with the Control group, mitochondrial morphology in the LPS group was smaller and fragmented. Mitochondrial morphology in the LPS + Rapa and Rapa groups was restored to an elongated shape (Figure 6C). These results indicate that promoting mitophagy alleviates LPS‐induced excessive mitochondrial fission.

We then explored the effect of mitophagy on mitochondrial function; mitochondrial MMP, mtROS, and ATP production levels were measured after LPS and rapamycin administration. Compared with the LPS group, there was increased MMP (*p* < 0.01), decreased mtROS levels (*p* < 0.05) and increased ATP production (*p* < 0.05) in the LPS + Rapa group (Figure 6D–F). These results suggest that promoting mitophagy can also improve mitochondrial function.

## 4 DISCUSSION

In the present study, anesthesia and surgery led to a decline in hippocampal‐related spatial learning and memory in aged rats. Both the MWM data in the current study and the previous Fear Conditioning Test results have confirmed the neurobehavioral changes.^29^ Exploratory laparotomy under sevoflurane anesthesia caused excessive mitochondrial fission in the hippocampal neurons of aged rats, which was accompanied by mitophagy inhibition. We speculate that the damaged mitochondria accumulated and were unable to be removed through mitophagy, resulting in mitochondrial dysfunction and ultimately dNCR in aged rats. An intraperitoneal injection of Mdivi‐1 (to interfere with Drp1) significantly ameliorated the levels of mitophagy and mitochondrial function and improved spatial learning and memory in aged rats after surgery. Furthermore, LPS‐induced HT22 cells also showed mitochondrial dysfunction accompanied by excessive mitochondrial fission and inhibition of mitophagy. HT22 cells transfected with siDrp1 had significant improvements in LPS‐induced mitochondrial dysfunction and mitophagy. Rapamycin improved mitophagy in HT22 cells and also improved LPS‐induced neuronal mitochondrial dysfunction and mitochondrial hyper‐division. Given that mitochondrial fission and mitophagy networks regulate and influence each other, our results suggest that mitochondrial dysfunction caused by disordered neuronal mitochondrial fission and mitophagy may be an important mechanism of abdominal surgery‐induced dNCR in aged individuals. By strengthening our knowledge of the pathogenesis‐related to mitochondrial dynamics based on mitochondrial fission and mitophagy, the establishment of dNCR model animals is expected to provide new intervention targets and ideas for the regulation of dNCR.

In the current study, aged rats with sevoflurane anesthesia had decreased spatial learning and memory after exploratory laparotomy; by contrast, no significant neurobehavioral abnormalities were observed after 30‐min exposure to 2.5% sevoflurane anesthesia only. This is consistent with the behavioral results of our previous study in which sevoflurane plus exploratory laparotomy, but not 2.5% sevoflurane anesthesia for half an hour, affected neurobehavior.^8^, ^30^ It may be that the traumatic stimulation caused by surgery, rather than by short‐term anesthesia exposure, leads to a series of changes in the central nervous system. Studies have confirmed that 1.4% isoflurane anesthesia for 2 h combined with laparotomy in aged mice results in a significant increase in mitochondrial fission in neurons of the hippocampus and prefrontal cortex 24 h after surgery; the mitochondria split from long rods into small round spheres and significantly increase in number.^15^ However, the potential molecular mechanism of increased mitochondrial fission was not explored in this previous study, and a causal relationship between increased mitochondrial fission and dNCR was not explored using interventions. In the present study, transcriptional levels of the mitochondrial fission proteins Drp1 and Fis1 and the fusion proteins OPA1, Mfn1, and Mfn2 were significantly increased in the hippocampus of aged rats on postoperative day 3. Nevertheless, only Drp1 increased among the fission and fusion proteins after surgery. By observing mitochondrial morphology with TEM, we identified an abnormal increase in mitochondrial fission, which we speculate was mainly caused by the significant increase of mitochondrial fission protein genes in the short term after surgery, with the increase of fusion protein genes compensating for these changes to achieve a balance between fission and fusion. However, such compensation was likely still unable to compensate for the damage caused by the increased fission proteins; the mitochondrial morphology we observed was therefore mainly characterized by increased fission. This is consistent with a previous study that reported significantly increased Drp1 and Mfn2 protein expression in the neuronal mitochondria of aged mice 1 day after surgery.^15^ We therefore speculate that abnormal increases in mitochondrial fission after surgery will inevitably have a serious impact on mitochondrial metabolism, mitochondrial function, and even the energy supply of neurons as a whole.

Drp1 is one of the most important proteins in the process of mitochondrial fission. Surgery activates Drp1 and increases mitochondrial fragmentation in the mouse hippocampus; fragmentation is also observed in primary neurons exposed to tumor necrosis factor.^16^ Previous studies have confirmed that mitochondrial fission can be divided into midzone fission and peripheral fission. Midzone fission is mainly related to actin‐mediated precontraction and MFF and regulates the proliferation process of healthy mitochondria, whereas peripheral fission is mainly regulated by the mitochondrial outer membrane protein Fis1, which selectively degrades mitochondria damaged by decreased membrane potentials or increased ROS during cell stress via lysosomes.^31^ The results of the present study suggest that surgery mainly affects the process of mitochondrial peripheral fission. However, whether it also affects midzone fission needs to be further studied.

The regulation of Drp1 phosphorylation can directly affect the process of mitochondrial fission.^32^ The phosphorylation of Drp1 at Ser637 reduces the recruitment of Drp1 from the cytoplasm to the mitochondria and reduces mitochondrial fission, thus promoting cell survival. By contrast, the dephosphorylation process can reduce the recruitment of Drp1 to the mitochondria and promote mitochondrial fission, thus increasing cell death. Unlike at Ser637, phosphorylation at Ser616 promotes mitochondrial fission whereas dephosphorylation inhibits it. In the present study, Drp1 phosphorylation at Ser616 was significantly increased in hippocampal neurons after exploratory laparotomy in aged rats, but there were no significant changes at Ser637. Together, these findings suggest that the phosphorylation of Drp1 at Ser616 promotes the mitochondrial fission process in the hippocampal neurons of aged rats.

Mdivi‐1 is a selective inhibitor of the mitochondrial fission protein Drp1. It can permeate the blood–brain barrier, thus inhibiting the excessive fission state of mitochondria in neurons.^33^ It has little effect on Drp1 activity and inhibits Drp1 phosphorylation at Ser616.^34^ Studies have confirmed that an intraperitoneal injection of 20 mg/kg Mdivi‐1 4 h before middle cerebral artery occlusion in mice can increase the extracellular adenosine content and thus play a neuroprotective role.^35^ Moreover, Mdivi‐1 alleviates nerve damage in glutamate neurotoxicity, oxygen, and glucose deprivation models, and ischemic brain injury by inhibiting Drp1.^36^ In addition, 50 μM Mdivi‐1 inhibits the LPS‐induced differentiation of macrophages into pro‐inflammatory cells by regulating neuronal mitochondria number and plays a neuroprotective role.^21^ Studies have also shown the excessive mitochondrial division of hippocampal neurons in rat models exposed to 2% isoflurane for 2 h; an intraperitoneal injection of 20 mg/kg Mdivi‐1 4 h before surgery can inhibit the excessive mitochondrial fission and reduce cell apoptosis.^20^ Together, these results suggest that the neuroprotective effect of inhibiting mitochondrial hyper‐fission in a dNCR model of aged rats with dual exposure to anesthesia and surgery is worthy of further study.

Because damaged mitochondria are selectively cleared through fission to maintain cell homeostasis, it remains unclear how mitochondrial fission and mitophagy are regulated in the dNCR model. Studies have confirmed that 5‐h exposure to 2% sevoflurane inhibits expression of the mitophagy regulatory protein Parkin in aged mice and thus inhibits the mitophagy process.^17^ However, the effect of exposure to both anesthesia and surgery on mitophagy in aged rats remains unclear, and the relationship between the two needs further elucidation. The current findings indicate that surgery damages mitochondria in aged rat hippocampal neurons; the damaged mitochondria are then released through the process of mitochondrial fission. However, surgery inhibits the mitophagy process so that damaged mitochondria cannot be removed by this specific process; in turn, the damaged mitochondria interact with healthy mitochondria to exchange genetic material, resulting in severe mitochondrial damage, mitochondrial dysfunction, and subsequent neuronal damage.

At a cellular level, we induced mouse hippocampal neuron HT22 cells with 1 μg/mL LPS for 24 h. We detected increased mitochondrial ROS levels, decreased MMP and ATP production, and mitochondrial dysfunction in these cells. The phosphorylation level of Drp1 at Ser616 increased; mitophagy was inhibited; LC3B and COXIV co‐localization decreased; and mitochondrial outer membrane, inner membrane, and matrix proteins increased; suggesting that mitophagy was inhibited. After Drp1 was knocked down, the autophagy flow of mitochondria was restored, LC3B and COXIV co‐localization increased, and the content of mitochondria‐related proteins decreased, suggesting that the inhibition of excessive mitochondrial fission improves mitophagy and mitochondrial function, promotes neuronal survival, and reduces the occurrence of dNCR. In this process, the inhibition of excessive mitochondrial fission can reduce the level of mitophagy inhibition, improve mitochondrial function, and improve hippocampus‐dependent spatial learning and memory function in aged rats. The promotion of mitophagy can also significantly improve excessive mitochondrial fission and improve mitochondrial function.

Previous studies have reported that p53 promotes the migration of Drp1 to mitochondria and increases mitochondrial fission in nonalcoholic liver disease models. P53 also inhibits the transcriptional expression of BCL2/adenovirus E1B 19 kDa protein‐interacting protein 3, leading to the inhibition of mitophagy and ultimately to mitochondrial dysfunction,^37^ which is consistent with the results of the present study. These authors also confirmed that melatonin can both inhibit mitochondrial fission and promote mitophagy, thus protecting liver function; they suggest that melatonin protects mitochondrial function through these two targets. The role of melatonin in the dNCR model is thus worthy of further study. In the hyperhomocysteinemia rat model, the mitophagy‐related proteins FUN14 domain containing 1 and LC3B and the mitochondrial fusion proteins Mfn1 and Mfn2 are down‐regulated, whereas expression of the mitochondrial fission protein Drp1 is up‐regulated. Moreover, the mitochondrial‐targeted H_2_S donor AP39 can regulate mitochondrial fission and fusion by activating mitophagy, thus reducing myocardial cell senescence and remodeling. PTEN‐induced putative kinase 1 promotes its binding to Parkin by Mfn2 phosphorylation, and Mfn2 deficiency in mice can prevent Parkin translocation to the mitochondria and inhibit mitophagy. The accumulation of mitochondria with abnormal morphology and function is an important mechanism of dilated cardiomyopathy.^38^ In acute kidney injury models, ischemia preconditioning activates Fundc1‐mediated mitophagy and reduces inflammation and kidney damage caused by ischemia–reperfusion injury; ischemia–reperfusion in Fundc1‐knockout mice leads to Drp1‐dependent excessive mitochondrial fission, mitochondrial dysfunction, and severe tubular cell death.^39^ In neonatal rat ventricular cardiomyocytes, high glucose‐enhanced mitochondrial fragmentation and reduced mitophagy flux are observed. Furthermore, the overexpression of Parkin increases mitochondrial fragmentation, whereas Drp1 overexpression accelerates mitophagy flux.^40^ Loss of Fis1 results in the accumulation of the Soluble NSF attachment Protein receptors protein syntaxin 17 in mitochondria and triggers mitophagy.^41^ These results suggest that there is a close relationship between mitochondrial fission/fusion and mitophagy, and indicates that they regulate each other and ultimately affect cell fate. Under the premise that mitophagy ability is normal, mitochondrial fission could trigger downstream mitophagy events. Our results suggest that surgery inhibits mitophagy ability so that it is insufficient to clear damaged mitochondria. As a result, damaged mitochondria accumulated and joined the mitochondrial circulation, resulting in the paralysis of mitochondrial system. The selective Drp1 inhibitor Mdivi‐1 may reduce mitochondrial fission and improve the outcomes caused by surgery, and thus restoring the mitophagy ability. Furthermore, ubiquitin‐dependent pink1–Parkin pathway and ubiquitin‐independent pathways such as Nip3‐like protein X (NIX), BCL2‐interacting protein3 (BNIP3), and FUN14 domain containing 1 (FUNDC1) may contribute to this phenomenon. The specific mechanism needs to be further studied. In the current study, we observed an ameliorative effect of reduced mitophagy inhibition on mitochondrial hyper‐fission at the in vitro level, but we did not conduct further verification at the in vivo level. Nevertheless, our previous studies in aged rats show that laparotomy significantly inhibits hippocampal mitophagy activity,^29^ which parallels the postoperative mitochondrial hyper‐fission seen in similar models.^15^ However, one limitation in the present study is that we did not verify the aforementioned regulatory effects of activated mitophagy on mitochondrial morphology using in vivo experiments. Because our previous study demonstrated that laparotomy surgery suppresses autophagy and causes hippocampus‐dependent cognitive deficits, we suppose that the regulation of mitophagy plays an essential role in dNCR. However, further investigation is required to elucidate the detailed interaction in the surgery‐challenged aged hippocampus.

To conclude, the roads to mitochondrial dysfunction in a rat model of postoperative dNCR appear extremely complex. The present study provides evidence for cognitive deficits in association with dysfunctional mitochondrial homeostasis and bioenergetics in hippocampal neurons after surgical trauma. Specifically, mitochondrial fission and mitophagy were closely coordinated and reciprocally interacted in the entire mitochondrial circulation network after surgery. Targeting mitochondrial fission and mitophagy in hippocampal neurons is therefore expected to be a new strategy for postoperative dNCR intervention.

## CONFLICT OF INTEREST STATEMENT

The authors declare that there is no conflict of interest.

## Supporting information


Data S1

## Data Availability

The data that support the findings of this study are available from the corresponding author upon reasonable request.
