# Supplementary material for: Reciprocal interaction between mitochondrial fission and mitophagy in postoperative delayed neurocognitive recovery in aged rats
Source: CNS Neurosci Ther. 2023 May 19;29(11):3322–38. doi: 10.1111/cns.14261 (PMC10580336; doi:10.1111/cns.14261)

Full unedited blot for Figure 2E-Drp1

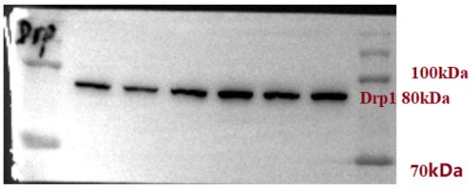

Full unedited blot for Figure 2E-Fis1

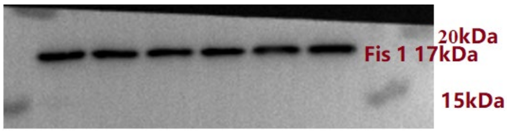

Full unedited blot for Figure 2E-OPA1

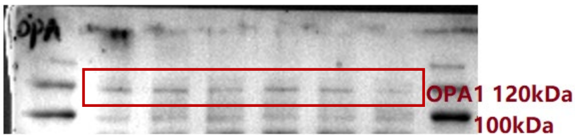

Full unedited blot for Figure 2E-Mfn1

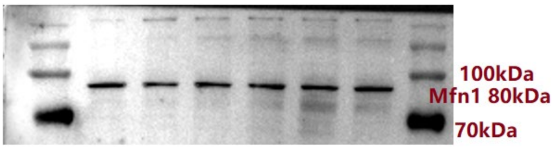

Full unedited blot for Figure 2E-Mfn2

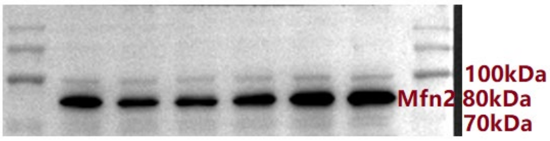

Full unedited blot for Figure 2E-β-actin

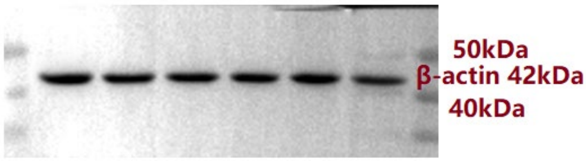

Full unedited blot for Figure 2G-p-Drp1(Ser616)

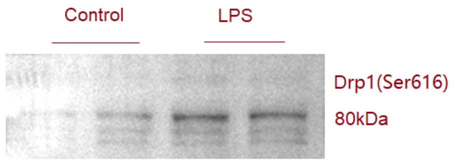

Full unedited blot for Figure 2G-Drp1

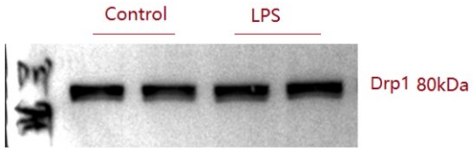

Full unedited blot for Figure 2G-β-actin

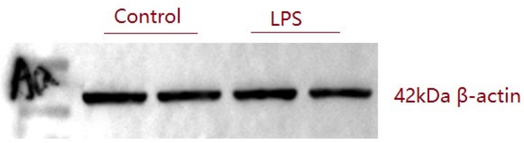

Full unedited blot for Figure2G-p-Drp1(Ser637)

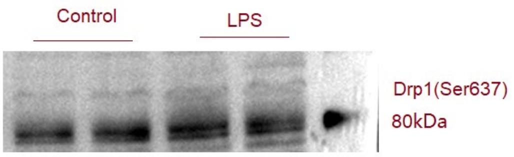

Full unedited blot for Figure3B-LC3B

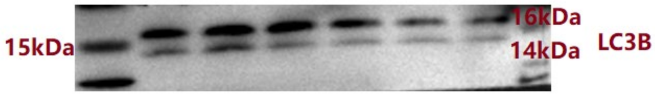

Full unedited blot for Figure3B-p62

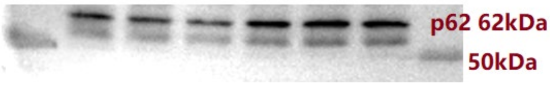

Full unedited blot for Figure3B-VDAC

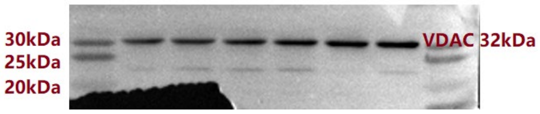

Full unedited blot for Figure3B-SOD2

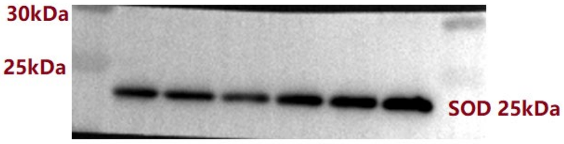

Full unedited blot for Figure3B-COXIV

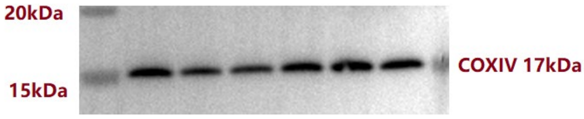

Full unedited blot for Figure3B-β-actin

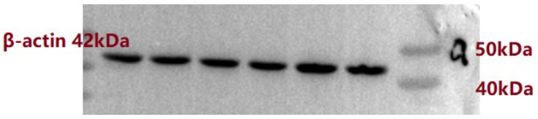

Full unedited blot for Figure3D-1μg/ml-COXIV

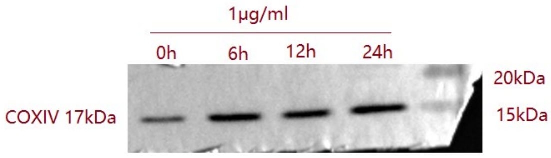

Full unedited blot for Figure3D-1μg/ml-LC3B

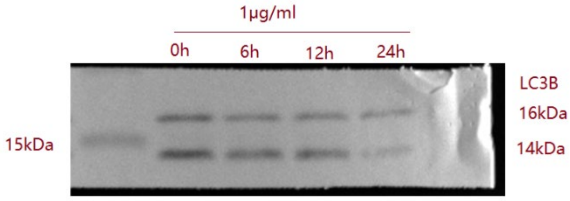

Full unedited blot for Figure3D-1μg/ml-p62

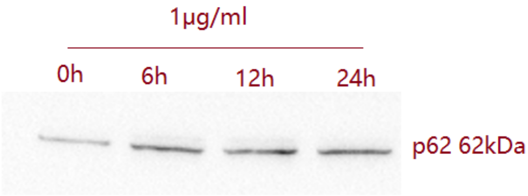

Full unedited blot for Figure3D-1μg/ml-SOD2

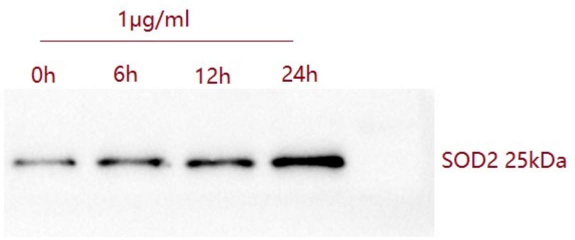

Full unedited blot for Figure3D-1μg/ml-VDAC

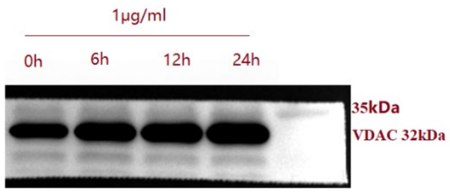

Full unedited blot for Figure3D-1μg/ml-β-actin

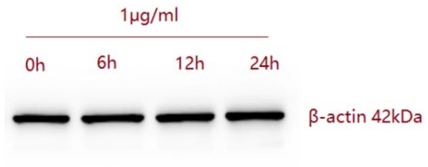

Full unedited blot for Figure3D-24h-COXIV

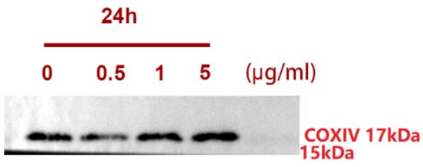

Full unedited blot for Figure3D-24h-LC3B

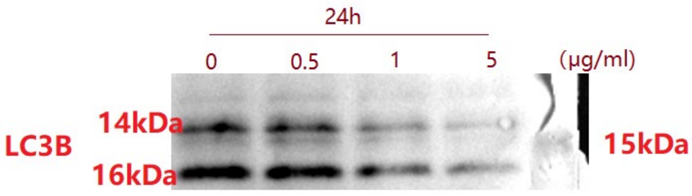

Full unedited blot for Figure3D-24h-P62

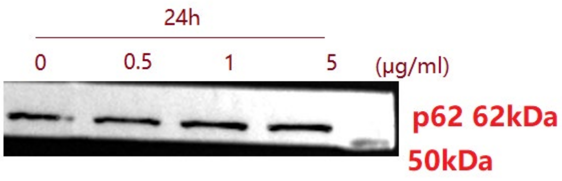

Full unedited blot for Figure3D-24h-SOD2

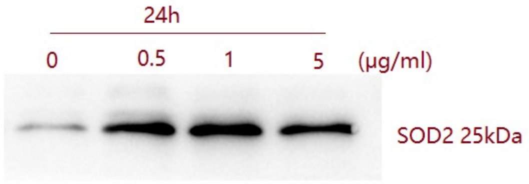

Full unedited blot for Figure3D-24h-VDAC

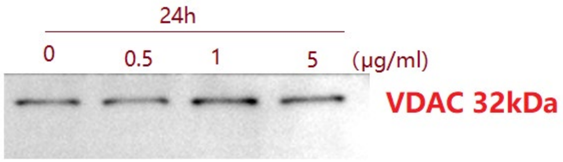

Full unedited blot for Figure3D-24h-β-actin

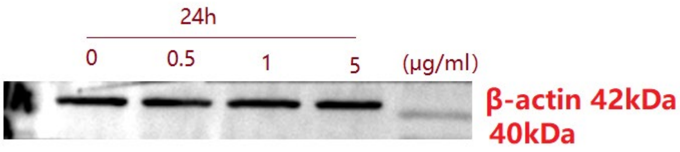

Full unedited blot for Full unedited blot for Figure5A-COXIV

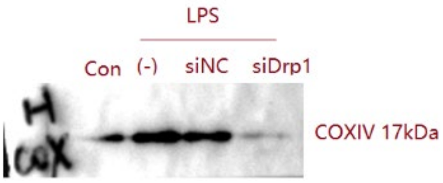

Full unedited blot for Figure5A-Drp1

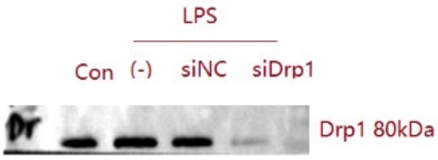

Full unedited blot for Figure5A-LC3B

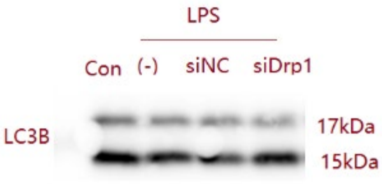

Full unedited blot for Figure5A-p62

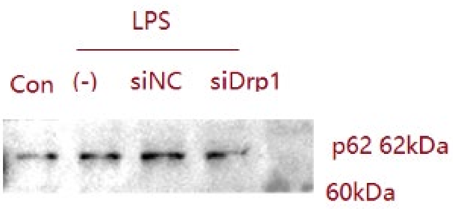

Full unedited blot for Figure5A-SOD2

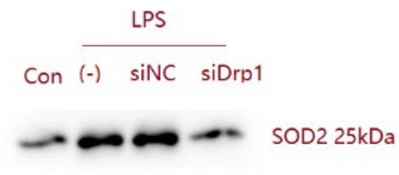

Full unedited blot for Figure5A-VDAC

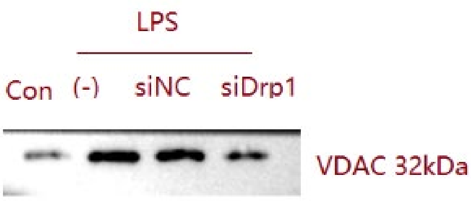

Full unedited blot for Figure5A-β-actin

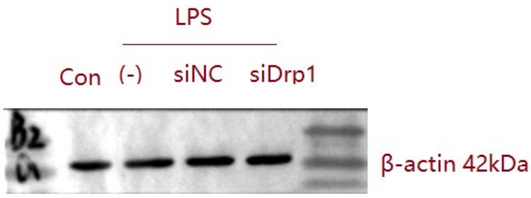

Full unedited blot for Figure5B-Drp1

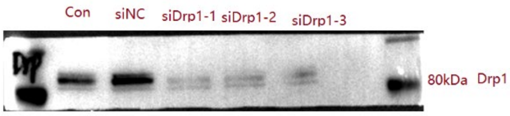

Full unedited blot for Figure5B-β-actin

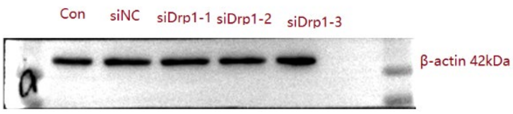

Full unedited blot for Figure6A-p-Drp1(Ser616)

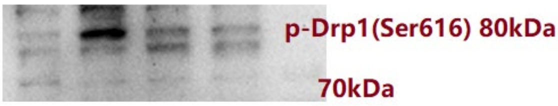

Full unedited blot for Figure6A-p-Drp1(Ser637)

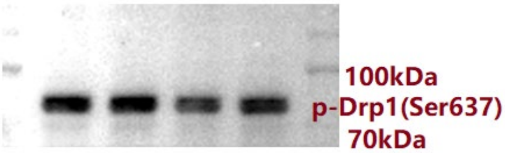

Full unedited blot for Figure6A-Drp1

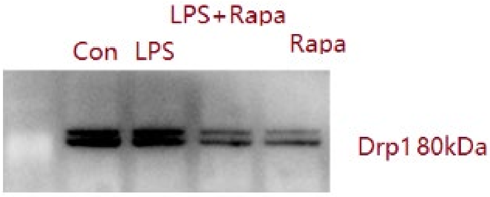

Full unedited blot for Figure6A-β-actin

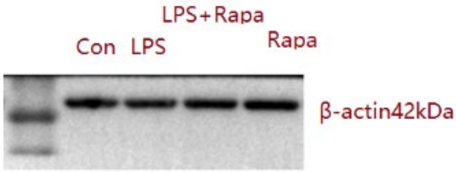

Supplement: Supplementary file 1 — Data S1 [file CNS-29-3322-s001.pdf]
